# Fabrication of Biomedical Ti-Zr-Nb by Reducing Metal Oxides with Calcium Hydride

**DOI:** 10.3390/jfb14050271

**Published:** 2023-05-13

**Authors:** Sergey Yudin, Ivan Alimov, Sergey Volodko, Alexander Gurianov, Galina Markova, Anatoly Kasimtsev, Tatiana Sviridova, Darya Permyakova, Evgeny Evstratov, Vladimir Cheverikin, Dmitry Moskovskikh

**Affiliations:** 1 Directorate, L.L.C. Metsintez, 300041 Tula, Russia; alimov.iwann@mail.ru (I.A.); volodko.sv@yandex.ru (S.V.); alex19021861@gmail.com (A.G.); metsintez@yandex.ru (A.K.);; 2 Mechanical Engineering and Materials Science Department, Tula State University, 300012 Tula, Russia; galv.mark@rambler.ru (G.M.); darya.per@gmail.com (D.P.); 3 Center for Project Activities of the Moscow Polytechnic University, Moscow Polytechnic University, 107023 Moscow, Russia; 4 Center of Functional Nano-Ceramics, National University of Science and Technology MISIS, 119049 Moscow, Russia; 5 Institute of New Materials and Nanotechnology, National University of Science and Technology MISIS, 119049 Moscow, Russia; 6 The Department of Surface Physics and Chemistry and Ultrafine Powder Materials, A. A. Baikov Institute of Metallurgy and Material Science of the Russian Academy of Sciences, 119334 Moscow, Russia; evev@imet.ac.ru; 7 Department of Physical Metallurgy of Non-Ferrous Metals, National University of Science and Technology MISIS, 119049 Moscow, Russia; cheverikin80@rambler.ru

**Keywords:** β-Ti alloy, Ti-Zr-Nb, calcium hydride synthesis, solid-state diffusion, reaction mechanism

## Abstract

In the present study, a powder of Ti-18Zr-15Nb biomedical alloy with spongy morphology and with more than 95% vol. of β-Ti was obtained by reducing the constituent oxides with calcium hydride. The influence of the synthesis temperature, the exposure time, and the density of the charge (TiO_2_ + ZrO_2_ + Nb_2_O_5_ + CaH_2_) on the mechanism and kinetics of the calcium hydride synthesis of the Ti-18Zr-15Nb β-alloy was studied. Temperature and exposure time were established as crucial parameters with the help of regression analysis. Moreover, the correlation between the homogeneity of the powder obtained and the lattice microstrain of β-Ti is shown. As a result, temperatures above 1200 °C and an exposure time longer than 12 h are required to obtain a Ti-18Zr-15Nb powder with a single β-phase structure and uniformly distributed elements. The analysis of β-phase growth kinetics revealed that the formation of β-Ti occurs due to the solid-state diffusion interaction between Ti, Nb, and Zr under the calcium hydride reduction of TiO_2_ + ZrO_2_ + Nb_2_O_5_, and the spongy morphology of reduced α-Ti is inherited by the β-phase. Thus, the results obtained provide a promising approach for manufacturing biocompatible porous implants from β-Ti alloys that are believed to be attractive candidates for biomedical applications. Moreover, the current study develops and deepens the theory and practical aspects of the metallothermic synthesis of metallic materials and can be compelling to specialists in powder metallurgy.

## 1. Introduction

Nowadays, pure titanium and Ti-Al-V alloys are widely used for elaborating bone implants for the human body [1]. In addition, shape memory alloys such as NiTi are also utilised for implants operating under severe loads in orthodontic treatments and bone plate applications [2]. However, these materials cannot respond to the high demands for biocompatibility and affinity in the mechanical behaviour of human bones due to higher stiffness and higher Young’s modulus (110–120 GPa for Ti-Al-V [3] and 70–80 for NiTi [4]). However, bone mineral, which is a component of bone tissue, possesses a Young’s modulus of 78–82 GPa. Considering the porous structure of human bones, Young’s modulus decreases to 10–22 GPa along a longitudinal direction and 5–13 GPa along a cross direction (cortical bone) [5]. This justifies the current demands in the development of biomedical alloys with similar mechanical behaviour to human bones. Moreover, the release of highly toxic vanadium and aluminium in the case of Ti-Al-V alloys and nickel in the case of NiTi alloys, in combination with high elastic properties, makes them unpromising materials for biomedical applications [6].

Efforts have been made to design new materials that do not contain highly toxic elements (V, Al, Ni, etc. [6]) and exhibit similar elastic behaviour to human bones. In this regard, the β-Ti alloys of the Ti-Zr-Nb system are attractive candidates for biomedical applications because they contain biocompatible and corrosion-resistant elements (Ti, Zr, Nb [6]) and demonstrate superelastic behaviour under applied loads owing to the thermoelastic β↔α” martensitic transformation, which occurs in a specific range of composition. As a result, the crystallographic strain of these alloys can approach 6% [7,8,9,10], which provides large recoverable deformations and a relatively low Young’s modulus. Moreover, constituent metals (Ti, Zr, and Nb) are shown to possess high biocompatibility and good acceptability [11,12]. According to [7], the Ti-18Zr-15Nb (% at.) alloy exhibits the best combination of mechanical and superelastic properties.

Nowadays, vacuum induction melting (VIM) and vacuum arc (re)melting (VAR) are the most common approaches for the fabrication of Ti-Zr-Nb alloys [7,8,13,14,15]. The main disadvantage of vacuum induction melting is the chemical liquation that takes place during solidification due to the different densities (Ti~4.5 g/cm^3^, Zr~6.45 g/cm^3^, Nb~8.57 g/cm^3^) and melting points of the constituent metals (Ti—T_m_ = 1670 °C, Nb—T_m_ = 2649 °C, Zr—T_m_ = 1855 °C). The significant chemical inhomogeneity of Nb is demonstrated in the Ti-18Zr-14Nb alloy after the first melting. Subsequent remelting leads to better homogeneity, but the goodness of elemental distribution is achieved after more than five remelts [13].

Recently, powder metallurgy (PM) has attracted particular interest as a promising production method for Ti-Zr-Nb [16,17,18,19] due to the possibility of obtaining porous structures with variable porosity, which leads to the better acceptability of the implant by bone tissue. In light of that, the uniform distribution of pores, open porosity, and a pore size in the range of 200–500 μm are crucial factors [20,21]. For example, in [16], the powders of Ti-Zr-Nb are manufactured by the hydration–dehydration method; then, the powders are mixed with ammonium bicarbonate and sintered at 1300 °C. Ammonium bicarbonate particles act as additional agents providing the desirable porosity by evaporating at sintering. The result is a structure with a high open porosity of 70% and a mean pore size of 260 μm which exhibits better acceptability than casting alloys. Furthermore, a decrease in Young’s modulus due to increasing porosity is an advantage.

In the patent [17], a permeable foam material is obtained from a Ti-(14–22)Nb-(6–18)Zr (% at.) spherical powder of 50 μm size and polymethyl methacrylate powder of 250 μm size by pressing under 150–200 MPa, followed by two-step sintering at 450 °C for 3 h and 1400 °C for 4 h. This technique results in the required porosity level with a pore size of 100–800 μm and a simultaneous decrease in Young’s modulus. However, the Ti-Zr-Nb powder is produced via the plasma rotating electrode process, which is a low-yielding, energy-consuming, and complicated piece of equipment; this is the main drawback of this method: the high cost of the Ti-Zr-Nb powder.

The classical powder metallurgy approach is used in [18]. The Ti-19Nb-14Zr (% wt.) powder alloy is fabricated by the reaction sintering of pure metals: Ti, Zr, and Nb. Authors take that into consideration for obtaining a single-phase product; the average size of the constituent metals must be smaller than 50 μm for Ti and 0.7 μm for Zr and Nb. On the other hand, the solid-state diffusion process, which governs the formation process of alloys, can cause the non-uniform distribution of elements and, consequently, the appearance of secondary phases.

In the present study, a Ti-18Zr-15Nb alloy is obtained using the metallothermic method. Its origin lies in the reduction of the mixture of TiO_2_, ZrO_2_, Nb_2_O_5_ by CaH_2_. The reduction agent in that reaction is Ca, which forms at the dissociation of CaH_2_. The adoption of CaH_2_ is attributed to its brittleness, which facilitates grinding and mixture preparation. The calcium hydride reduction process occurs most actively in the temperature range of 900–1200 °C, which is lower than the melting points of the constituent metals and the Ti-18Zr-15Nb alloy. This prevents the element segregation that occurs during the solidification of the casting alloys. The calcium hydride process has proven itself to produce intermetallics [22], ultra-high temperature ceramics based on Hf [23], and ultrafine and nano-sized powders of titanium and zirconium carbides [24]. The given powders exhibit high homogeneity and, in the case of HfCN, TiC, and ZrC, possess perfect crystal structures. Additionally, Ti-Nb alloys are fabricated using the calcium hydride method, and the futures of β-phase decomposition have been previously studied [25].

Therefore, there is reason to believe that the calcium hydride method can produce a Ti-18Zr-15Nb powder with the desired chemical and phase compositions. However, there has been no systematic research on the influence of the processing parameters, such as temperature, exposure time, and density of a charge. Thus, the present study aims to investigate the effect of the processing parameters on the kinetics and mechanism of calcium hydride synthesis of the Ti-18Zr-15Nb powder alloy.

## 2. Materials and Methods

To obtain Ti-18Zr-15Nb by calcium hydride synthesis, TiO_2_ (purity of ≥95% wt.), ZrO_2_ (≥99.5% wt.), Nb_2_O_5_ (≥99.5% wt.), CaH_2_ (≥96.0% wt.) were mixed in a roll ball mill for 40 min at 60 rpm. The mass ratio of the constituent metals, counting their content in the oxides, was taken as follows: Ti—51.38% wt., Zr—26.30% wt., Nb—22.32% wt. The mixture was loaded into a steel tube. After that, the mixture was pressed at a density of 1.4 and 1.6 g/cm^3^ under 0.7 and 56 MPa. The upper stress value is constrained by the limit of operation load for a hydraulic pressing machine because of the large cross section (10.7 cm^2^) of the charge (the mixture of the oxides and calcium hydride). Further, the steel tube with the charge was set in a container made from heat-resistant chromium steel and tightly closed with a steel cover. Air was evacuated from the container at a pressure of 1.33 Pa, and Ar was pumped into it. Then, the calcium hydride synthesis was carried out in a pit electric resistance furnace. In the first assumption, the calcium hydride synthesis of the Ti-18Nb-15Zr alloy can be described by the following reaction:

0.67 TiO_2_ + 0.09 Nb_2_O_5_ + 0.15 ZrO_2_ + 2.09 CaH_2_ → Ti_0.67_Nb_0.18_Zr_0.15_ + 2.09 CaO + 2.09 H_2_↑ (1)

**Figure 1:**
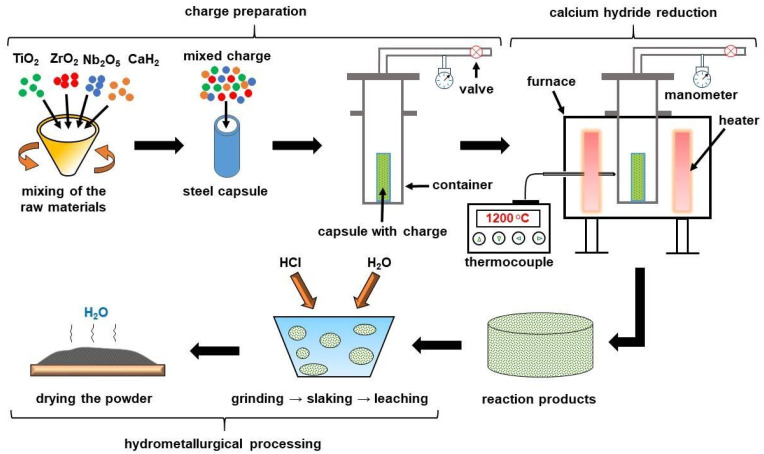
Technological route of the fabrication of the Ti-18Zr-15Nb powder alloy.

In this reaction, CaO is a by-product. Its removal from the reaction product consists of the following iterations: (1) grinding the synthesis product; (2) loading the ground powder into a glassware and adding water; (3) mixing and making a pulp state; (4) adding HCl and mixing; (5) washing the operation volume with water several times. During HCl-treatment, the pH value was kept at around 2–3, and then it was increased to 7 while being washed. Eventually, the powder was placed in a dryer, held for three hours at 60 °C, and sieved. The schematic representation of the calcium hydride process is described in Figure 1.

To examine the structure of the synthesized powders, X-ray analysis was carried out using a DRON-3 diffractometer (Burevestnik, Russia) with monochromatic CuKα radiation. The phase composition and weight fractions of each phase were estimated in a specific program using the Rietveld refinement method [26]. Relative errors of phase fractions and lattice constants did not exceed 5 and 0.15%, respectively. To avoid the influence of dissolved hydrogen on the XRD profiles, the powders were annealed at 700 °C for 20 min under a vacuum of 6.7 × 10^−3^ Pa.

The structure of the powders was studied using scanning electron microscopy JSM7600F (JEOL, Tokyo, Japan) with an energy dispersive spectrometer EDX (Oxford Instruments, Abingdon, UK).

Microstrain values were calculated using the Rietveld method with the following equation using the pseudo-Voight profile function to process both experimental and standard profiles:

(2) $$e_{i}=\frac{{\left[\left(U_{i}-U_{std}\right)-\left(W_{i}-W_{std}\right)\right]}^{0.5}}{A}$$

where *U* and *W* are peak profile parameters for the Caglioti function of the sample and standard, and *A* is a constant. To investigate instrumental broadening, coarse-grained germanium stands as standard. The mean error value for the microstrain is calculated as the square root from the variance, which is estimated using the following equation:

(3) $$\sigma^{2}=\frac{A^{2}\times [\sigma^{2}\left(U_{i}\right)+\sigma^{2}\left(U_{std}\right)+\sigma^{2}\left(W_{i}\right)+\sigma^{2}\left(W_{std}\right)]}{4[\left(U_{i}-U_{std}\right)-\left(W_{i}-W_{std}\right)]}$$

## 3. Results and Discussion

There are two crucial parameters which strongly influence the phase composition and homogeneity of the final powder of the calcium hydride process: temperature and exposure time. Additionally, the density of a green body, which can also be called a charge, is a crucial parameter too. In terms of the density of the charge, we assume the density of the mixture prepared for the subsequent synthesis. Commonly, in the calcium hydride process, a charge is not subjected to pressing with a hydraulic press machine and can only be hand-pressed. However, to investigate the effect of pressing on the yield of the product, the charge in the present study is pressed under 0.7 and 56 MPa which results in 1.4 and 1.6 g/cm^3^ charge densities, respectively. The higher charge density is expected to provide a better interaction of reduced metals and promote the synthesis.

The design of experiments has been applied to systematically investigate the effect of the processing parameters on the yield of the β-phase. The design matrix is represented in the Supplementary File. The quantitative variables, such as processing temperature and exposure time, are varied from 900 to 1200 °C and from 0 to 6 h, respectively. The chosen ranges are constrained by the heat resistance of the container and the low diffusion activity of refractory elements below 900 °C.

**Figure 2:**
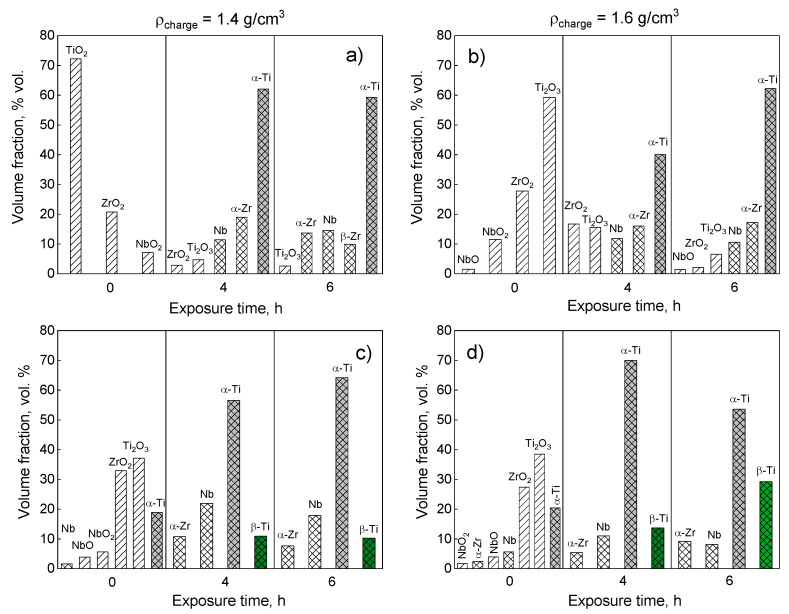
The phase composition of the Ti-18Zr-15Nb powder alloy after reduction at 900 °C (**a**,**b**) and 1000 °C (**c**,**d**) with pressing of the charge using different methods.

Figure 2 depicts the histograms demonstrating the phase composition of the powders synthesized at 900 and 1000 °C with 1.4 and 1.6 g/cm^3^ charge densities. Previously, it was shown that if liquid calcium appears above 840 °C, the reduction process of oxides actively proceeds [22]: CaH_2_ starts to decompose, and Ca melts in the temperature range of 839–900 °C depending on the hydrogen content [27]. Subsequently, below 1000 °C, oxide phases can remain in the product, especially after short exposure times. Figure 2a,b show that oxide phases remain after the reduction at 900 °C and for all exposure times. Controversially, at a reduction temperature of 900 °C (Figure 2a,b), the amount of the oxides is relatively higher in the powder with the higher charge density. Moreover, the formation of the β-phase did not occur at 900 °C, including the prolonged exposure time, and the final products consisted only of pure metals, which may stem from the low diffusion activity of these elements. An increase in the reduction temperature up to 1000 °C accelerates reduction reactions promoting the reduction of oxides; this results in the absence of oxide phases for all exposure times despite the regimes conducted without exposure. Furthermore, the β-phase appears (less than 5% vol.) at 1000 °C for a 2 h holding time (see Table S1 in the supplementary file), and its amount slightly increases with the increasing exposure time. The formation of the β-phase occurs through the dissolution of Nb in Ti, since Nb is a β-stabilizing element for titanium alloys that prevents the transformation from β-Ti, which is stable at processing temperatures, to α-Ti, which is stable at room temperature [28]. However, the main products at room temperature are α-Ti, α-Zr, and Nb for all exposure times at 1000 °C (Figure 2c,d).

Several XRD patterns of the Ti-18Zr-15Nb powders synthesized at different modes and demonstrating various phase compositions (oxides, pure metals, β-Ti, etc.) are demonstrated in the Supplementary file in Figure S1. Phase compositions are listed in Table S2.

**Figure 3:**
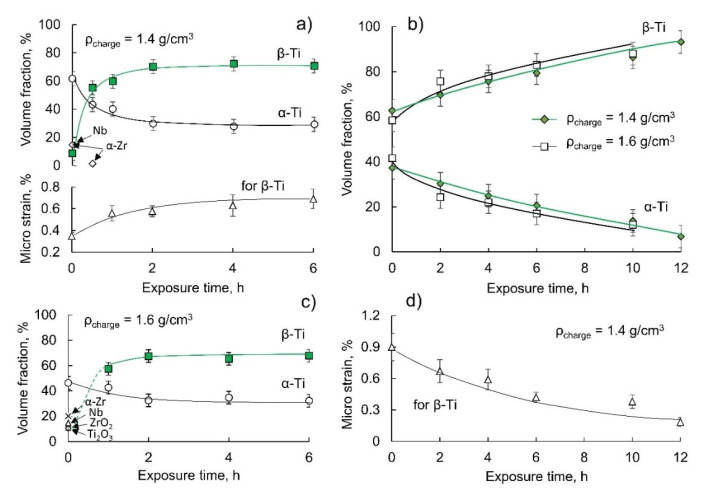
The effect of exposure time on the phase composition and the lattice strain of β-phase of Ti-18Zr-15Nb powder synthesized at 1100 °C (**a**,**c**) and 1200 °C (**b**,**d**) with different charge density.

Figure 3 demonstrates the influence of the processing temperature and charge density on the phase composition of the powders synthesized at 1100 and 1200 °C and the lattice microstrain of the β-phase. The α-Ti dominates in the structure of the powder obtained at 1100 °C with null exposure time (Figure 3a) for a charge density of 1.4 g/cm^3^. α-Zr and Nb still remain, and a small amount of the β-phase was observed. However, the amount of β-phase rapidly increased 7 times from 10 to 70% vol. within the first 2 h of the exposure due to the dissolution of Zr and Nb in Ti, resulting in β-Ti, and stays almost constant for up to 6 h.

In multi-component systems, such as Ti-Zr-Nb [29], the diffusion rate depends on chemical composition, particularly on a composition gradient according to Fick’s second law. Therefore, probably in our case, after 2 h of exposure at 1100 °C, a dynamic quasi-equilibrium is reached between the composition of the phases within the particles under which diffusion begins to slow down. To accelerate solid-state diffusion, an extra-driven force provided by an increase in the processing temperature that has been observed in our research is needed; this will be discussed later (Figure 3b).

The interesting effect has been taken into consideration for the influence of hydraulic pressing on the phase composition of the powder obtained at 1100 °C (Figure 3c). For null exposure time, the main phase is α-Ti with Ti_2_O_3_, ZrO_2_ (~10% vol. for each oxide) and metals α-Zr, Nb (15–20% vol. for each) remain. β-Ti is not observed; however, it forms for exposure times longer than 1 h at 1100 °C and becomes dominant. An increase in exposure time up to 6 h has shown to slightly affect the β-phase amount; that is, it has already reached the maximum at a temperature of 1100 °C after a 2-h exposure.

A 55–65% vol. β-Ti forms during the heating to 1200 °C, and monotonically grows with increasing exposure time from 0 to 12 h. However, after prolonged exposure, the powder still has α-Ti, and a single β-phase structure was not observed according to the XRD profiles (Figure 3b). Further, an increase in exposure time provides the formation of a ~95% vol. of β-Ti. The approximation and interpolation of the relationship between the β-phase amount and the exposure time for both charge densities yields the value of the dwelling time to obtain ~100% of β-Ti in the structure, which is within 14.5–15 h. On the other hand, the single-phase structure should be formed at consolidation, which includes the vacuum sintering at elevated temperatures for several hours. To summarize, the charge pressing does not affect the β-phase formation noticeably. We assume that such a low increase in the green body’s density by altering the load from 0.7 to 56 MPa (~14%) does not affect the growth kinetics of the β-phase.

The lattice microstrain of the β-phase has been analysed to explain the stagnation in β-phase growth at 1100 °C. Moreover, the assumption that crystallite size does not affect the line width is adopted considering the powder size, which is more than 2 μm on average. Therefore, we are only counting the impact of chemical inhomogeneity on the strain of a crystal lattice, which results in the line broadening. Thus, the lattice strain is defined by different covalent atomic radii of Ti, Zr, and Nb, which are 1.467, 1.597, and 1.456 Å, respectively, in an approximation of the BCC lattice with a coordination number of 12 in accordance with Poling [30] and their distribution uniformity.

The lowest value of the microstrain was observed with a short exposure time at 1100 °C (Figure 3a). This indicates the start of β-phase formation, and inhomogeneity is still insufficient. With increasing exposure time, the microstrain value increases due to the saturation of the β-phase by Nb and Zr, which brings a non-uniform distribution of those elements within the phase. The further increase in the exposure time promotes the diffusion of those elements and probably reaches its maximum. Possibly, a significant increase in the exposure time at 1100 °C can cause the further interaction of constituents, and upon the formation of the β-phase, the homogenization would lead to a parabolic character of the lattice strain curve with its drop to lower values, as was further shown at 1200 °C.

At 1200 °C and null exposure time, the β-phase exhibits the highest microstrain value, which is explained by the non-uniform distribution of constituents due to diffusion in β-phase. By increasing the exposure time, the microstrain decreases and eventually drops to 0.18%; this is a typical value for crystalline annealed alloys. The drop in the microstrain can be explained by the following: the small particles, titanium in our case, are saturated with other elements faster than large particles, and homogenization occurs within those small particles, causing the drop in the microstrain. In other words, two different competing processes take place. On the one hand, the saturation of Ti with Nb and Zr occurs to critical concentration, causing β-phase formation: the β-solid solution is not homogeneous, which alters the microstrain. On the other hand, high processing temperatures cause a drop in the microstrain via homogenization processes. The first case prevails at 1100 °C whereas the second at 1200 °C.

Simultaneously, the diffusion proceeds in large particles followed by homogenization, resulting in an additional decrease in the microstrain. The greater number of particles where homogenization proceeds, the lower the microstrain value. Logically, this explanation ought to be followed by a monotonical increase in β-phase amount, which has been observed in our case (Figure 3d). Similar processes proceed at 1100 °C, but prolonged exposure is required.

**Figure 4:**
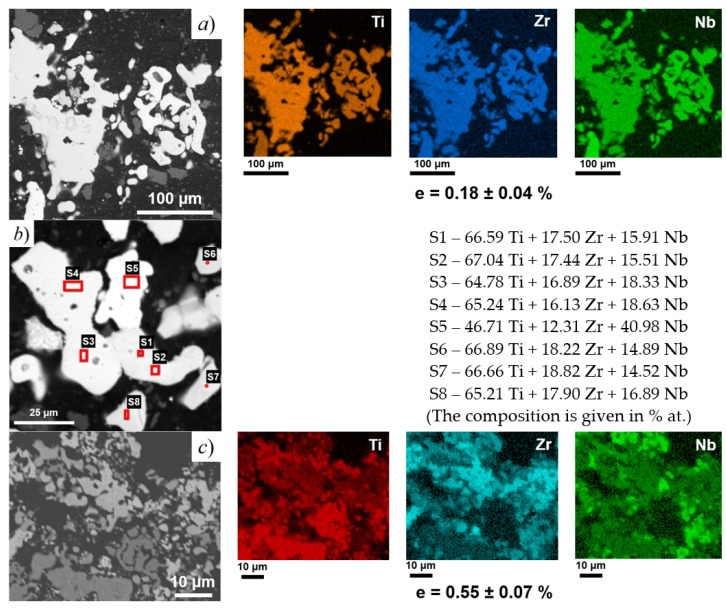
Results of EDS analysis of the powders (ρ_charge_ = 1.4 g/cm^3^) synthesized under different modes: (**a**,**b**) 1200 °C, 12 h and (**c**) 1100 °C, 0 h.

Figure 4 shows the comparison of the element distributions of the powders exhibiting different microstrain values. According to the low microstrain value, the powder after 12 h at 1200 °C should exhibit relatively uniform element distribution within the particles; this is shown in Figure 4a. Additionally, the EDS analysis (Figure 4b) revealed the uniform distribution of Zr. The variation in Zr content does not exceed 1% at. related to the nominal composition of the alloy, but Nb is distributed less homogeneously. The variation in Nb content is established as (±2% at.), which is most likely due to its highest melting point among constituents and, subsequently, low diffusivity.

However, the constituents are non-uniformly distributed in the powder synthesized at 1100 °C, 0 h, where severe lattice microstrain is observed. Therefore, a 12 h exposure at 1200 °C is required for decent quality in the final product.

The abnormal growth kinetics of the β-phase at a temperature above 1100 °C (Figure 3a) is probably related to the heat released during the reduction reaction. The profound exothermic effect can be observed in the reduction of TiO_2_ by calcium at temperatures 1050–1100 °C. The exothermic type of reaction can supply auxiliary heat to the charge promoting β-phase formation. Therefore, the heat released can additionally increase the temperature in the charge volume, contributing to β-phase formation. It is well known that within the self-combustion synthesis method, there is a special approach that is suitable for obtaining alloys based on solid solutions where the reaction exhibits a low-exothermic effect. If not enough heat is revealed during the reaction, an additional heat source should be added. For example, the reaction Ti + C → TiC + Q↑ stands as an additional heat supplier during the fabrication of the composite based on the CoCrFeNiMn alloy [31,32]. While TiC forms, the release of heat can melt the alloys’ components, providing the rapid formation of the product. Since the β-solid solution is more likely to form through solid-state diffusion, it cannot bring enough heat to provide the rapid formation of the β-phase. In our case, an additional heat source could be the reaction TiO_2_ solid + 2Ca liquid → Ti solid + 2CaO solid + Q↑ or the similar reactions of the reduction Zr and Nb by Ca. 

**Figure 5:**
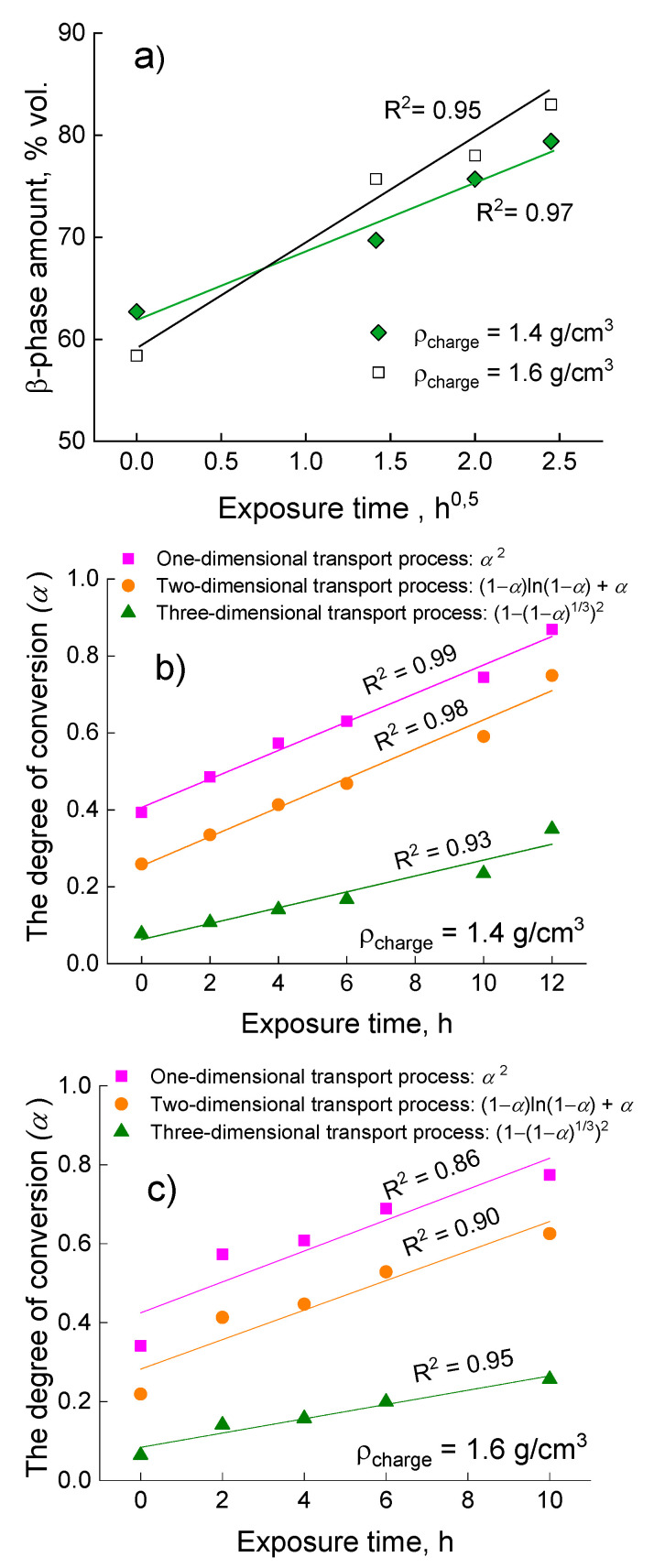
The experimental data representation for powders obtained at 1200 °C: β-phase amount versus the square root of exposure time (**a**); relationship between degree of conversion *a* and exposure time (**b**,**c**).

To prove the hypothesis that β-phase formation is governed by solid-state diffusion, the kinetics of β-phase growth at 1200 °C is analysed via graphical methods, as shown in Figure 5. The nucleation is believed to be controlled by solid-state diffusion if the increase in the phase amount is proportional to the square root of exposure time [33,34]. Figure 5a shows the straight-line relationship between the β-phase amount and the square root of the synthesis time, which means that the β-phase forms by the solid-state diffusion mechanism. Delving into the diffusion mechanism, some other diffusion models can be discussed. Šesták J. and Berggren G. summarize and describe a variety of kinetics laws for solid-state reactions [35] which can be utilized in our case. Figure 5b,c show the relationship between *a* (degree of conversion—the volume fraction of a target phase) and an exposure time for 1200 °C processing temperature. There are three different laws for interpretating the transfer mechanism: one-, two-, and three-dimensional transport processes. By a one-dimensional process is meant the mono-side diffusion in an assumption of a flat continuous surface, which similar to that which is observed in diffusional couples. A two-dimensional mechanism implies the diffusion of atoms on a particle’s surface. A three-dimensional mechanism corresponds to volume diffusion in the assumption of a spherical shape. It has been shown that for powder with a charge density of 1.4 g/cm^3^, β-phase formation is prone to two different mechanisms: one- and two-dimensional transfer mechanisms, which can proceed simultaneously or one after the other (R^2^ = 0.98–0.99). In the case of the powder with a charge density of 1.6 g/cm^3^, three-dimensional transportation fits the experimental data more accurately (R^2^ = 0.95) (Figure 5c), probably because pressing provides tighter contact between constituents.

The crucial information enlightening the formation mechanism can be given by the morphology of synthesized powders. For example, components that have high or non-limited solubility in calcium melt are believed to form powders with a shape close to globular or spherical [22], which is observed for NiAl and Ni_3_Al intermetallic compounds. Otherwise, the particles possess a spongy morphology if the solubility of constituents is negligibly low, as observed for Cr_2_Ta [36].

In our case, the alloy consists of elements which almost do not dissolve in the calcium melt [22,37,38]. Generally, calcium hydride powders are characterized by a complicated spongy morphology [39,40,41]. Subsequently, to retain the same morphology while the alloy forms, the interaction of constituent elements should be of solid-state type.

**Figure 6:**
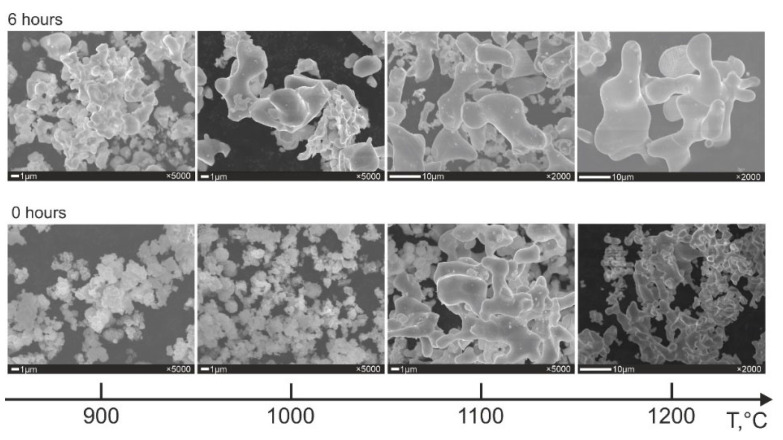
The evolution of the powder morphology of the Ti-18Zr-15Nb alloy (ρ_charge_ = 1.4 g/cm^3^).

Figure 6 shows the influence of the reduction temperature and the exposure time on the powder morphology for 1.4 g/cm^3^ charge density. It has been shown that the morphology of the powders obtained is similar to the morphology of reduced Ti, Nb, and Zr (Figure S2) which have a spongy morphology. In turn, a sponge is represented by smaller sintered particles.

The powder synthesized at 900 °C and null exposure time consists of particles similar to initial the TiO_2_, ZrO_2_, and Nb_2_O_5_. This agrees with the results of the X-ray analysis (Figure 2a) where the presence of oxides is shown; that is, the reduction process has not yet been finished. With an increase in the exposure time up to 6 h at 900 °C, the powder morphology drastically changes: the particles become more coarsened, and there are small particles present in the structure which have not been involved in the agglomeration due to the relatively low synthesis temperature. An increase in reduction temperature up to 1000 °C without exposure does not bring noticeable changes to the powder morphology despite the fact that 30% vol. of α-Ti and Nb, approximately, have already formed. After 6 h of exposure at 1000 °C, the oxides disappear (Figure 2c), and only reduced metals and a small amount of β-Ti are observed in the structure, resulting only in spongy particles of different shapes and sizes.

The particles of the powder synthesized at 1100 °C for 0 and 6 h are similar and consist of large particles with smaller particles on their surface. It should be noticed that with an increase in exposure time from 0 to 6 h at 1100 °C, the volume ratio α-Ti/β-Ti becomes reciprocal; that is, β-Ti starts to dominate. However, the powder morphology does not change. In other words, from the point of view of morphology, α-Ti and β-Ti are undistinguished, which emphasizes the solid-type interaction reaction of reduced metals.

After reduction at 1200 °C, regardless of the exposure time (Figure 6), relatively large spongy agglomerates of the β-phase are formed. As in the case of 1100 °C, the amount of β-phase increases due to a decrease in the amount of α-phase without any notable changes in the particle morphology with an increase in the exposure time at 1200 °C.

The regression analysis is applied to quantitatively evaluate the impact of each processing parameter. Using Minitab 21 statistical software, the forward selection with validation method was used, which resulted in the following regression model:

(4) $${Ti}_{\beta }=-234.7+0.2588\;Temperature\left({}^{\circ}C\right)+1.095\;Exposure\left(h\right).$$

**Figure 7:**
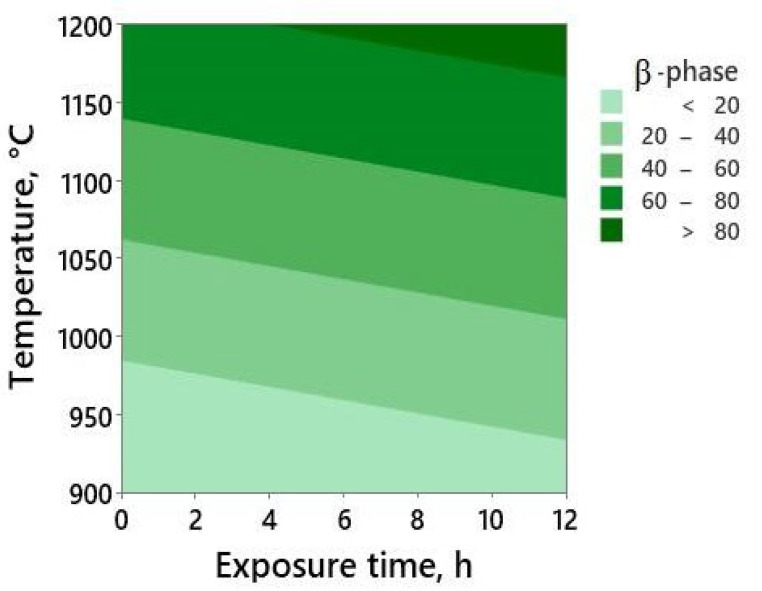
The volume fraction of β-Ti as a function of processing temperature and exposure time.

As shown in the regression model, the pressing is excluded from the model because it has a low significance for the *p* value = 0.1 set for the current regression analysis. *p* values for the temperature predictor and constant are less than 10^−3^, but it is quite high and estimated as 0.065 for the exposure-time predictor. This can be taken in the model only for a confidence level of 90%. The variance inflation factors for both models’ parameters are 1.03. This is close to 1, and it means that there is no correlation between them. Moreover, the standardized residuals obey a normal distribution law (Figure S3), which, with the aforementioned parameters, reveal the validity of the model. F values, which show the impact of each predictor, are 197.49 and 3.59 for the temperature and the exposure, respectively. Subsequently, the temperature contributes to the model more significantly than the exposure. The adjusted R-squared for the elaborated model is 83.3%. This value is considerably high, and it means that 83.3% of the variation in the β-phase amount can be described by the variation in temperature and exposure in the model, counting the impact of the two predictors in the model. To visually demonstrate the elaborated model, the processing map as a contour plot is shown in Figure 7.

## 4. Conclusions

In the present study, the influence of the processing parameters (reduction temperature, exposure time, a density of the charge) of the calcium hydride reduction process on the phase composition and the powder morphology of the powder Ti-18Zr-15Nb β-alloy were studied. The formation of the β-phase is governed by solid-state diffusion of reduced metals into Ti particles. The regression model of the calcium hydride synthesis of the Ti-18Zr-15Nb alloys was elaborated. The influence of each factor was demonstrated: the most crucial factor is the temperature followed by the exposure time. However, the pressing of the charge is insignificant and is not included in the regression model. The experimental data have shown that a yield of homogeneous β-Ti phase higher than 95% vol. can be reached under 1200 °C and 12 h of exposure. The synthesized powders possess a spongy morphology, and β-Ti inherits the morphology of α-Ti which proves the solid-state reaction mechanism. In turn, the spongy morphology of the calcium hydride powders can be a crucial factor when it comes to the production of high-porous structures with great acceptability and excellent superelastic behaviour, which provides a promising method for bone implant fabrication.

## Data Availability

Not applicable.

## Supplementary Materials

The following supporting information can be downloaded at: https://www.mdpi.com/article/10.3390/jfb14050271/s1. Figure S1: Experimental and calculated XRD patterns of calcium-hydride powders synthesized at various temperatures and exposure: *a*—900 °C, 0 h; *b*—1000 °C, 0 h; *c*—1000 °C, 6 h; *d*—1200, 4 h; *f*—1200, 12 h. *a*, *c*, *d*, *f—*(ρ_charge_ = 1.4 g/cm^3^); *b*—(ρ_charge_ = 1.6 g/cm^3^); *R_w_—weighted average Bragg R-factor*; Figure S2: Typical morphology of Ti, Zr, and Nb reduced by calcium hydride; Figure S3: Normal probability plot of standardized residuals for the elaborated model. Table S1: Design matrix; Table S2: Several structural parameters of the phases, which Bragg positions are shown in Figure S1.
