# Supplementary material for: Fabrication of Biomedical Ti-Zr-Nb by Reducing Metal Oxides with Calcium Hydride
Source: J Funct Biomater. 2023 May 13;14(5):271. doi: 10.3390/jfb14050271 (PMC10219185; doi:10.3390/jfb14050271)
Supplement: Supplementary file 1 [file jfb-14-00271-s001.zip › jfb-2367395-supplementary.pdf]

SUPPLEMENTARY MATERIAL

Fabrication of Biomedical Ti-Zr-Nb by Reducing Metal Oxides with Calcium Hydride

Sergey Yudin [1,2,3,*], Ivan Alimov [1,2], Sergey Volodko [1,4], Alexander Gurianov [1,2], Galina Markova [2], Anatoly Kasimtsev [1,2], Tatiana Sviridova [1,5], Darya Permyakova [2], Evgeny Evstratov [6], Vladimir Cheverikin [7] and Dmitry Moskovskikh [4,*]

[1] Directorate, L.L.C. Metsintez, Tula 300041, Russia; alimov.iwann@mail.ru (I.A.); volodko.sv@yandex.ru (S.V.); alex19021861@gmail.com (A.G.); metsintez@yandex.ru (A.K.); tim-17@yandex.ru (T.S.)

[2] Mechanical Engineering and Materials Science Department, Tula State University, Tula 300012, Russia; galv.mark@rambler.ru (G.M.); darya.per@gmail.com (D.P.)

[3] Center for Project Activities of the Moscow Polytechnic University, Moscow Polytechnic University,
Moscow 107023, Russia

[4] Center of Functional Nano-Ceramics, National University of Science and Technology MISIS, Moscow 119049, Russia

[5] Institute of New Materials and Nanotechnology, National University of Science and Technology MISIS, Moscow 119049, Russia

[6] The Department of Surface Physics and Chemistry and Ultrafine Powder Materials, A. A. Baikov Institute of Metallurgy and Material Science of the Russian Academy of Sciences, Moscow 119334, Russia; evev@imet.ac.ru

[7] Department of Physical Metallurgy of Non-Ferrous Metals, National University of Science and Technology MISIS, Moscow 119049, Russia; cheverikin80@rambler.ru

* Correspondence: sergey-usn@mail.ru (S.Y.); mos@misis.ru (D.M.)

Table S1. Design matrix.

Temperature, °C	Exposure, h	β-Ti, % vol.	Pressing method*
900	0.0	0.0	0
900	2.0	0.0	0
900	4.0	0.0	0
900	6.0	0.0	0
900	8.0	0.0	0
1000	4.0	10.9	0
1000	6.0	10.3	0
1050	4.0	53.8	0
1050	6.0	50.5	0
1100	0.5	55.2	0
1100	1.0	59.8	0
1100	2.0	70.2	0
1100	4.0	72.2	0
1100	6.0	70.7	0
1150	4.0	56.3	0
1150	6.0	59.4	0
1200	0.0	62.7	0
1200	2.0	69.7	0
1200	4.0	75.7	0
1200	6.0	79.4	0
1200	10.0	84.2	0
1200	12.0	93.2	0
1100	0.5	55.2	0
1100	1.0	59.8	0
1100	2.0	70.2	0
1150	4.0	56.3	0
1150	6.0	59.4	0
1200	10.0	84.2	0
1200	12.0	93.2	0
1000	4.0	13.7	1
1000	6.0	29.2	1
1050	4.0	62.2	1
1050	6.0	55.4	1
1100	1.0	57.4	1
1100	2.0	67.5	1
1100	4.0	65.3	1
1100	6.0	67.8	1
1150	4.0	59.9	1
1150	6.0	62.7	1
1200	0.0	58.4	1
1200	2.0	75.7	1
1200	4.0	78.0	1
1200	6.0	83.0	1
1000	0.0	2.9	1

* – 0 – hand pressing of the charge; 1 – hydraulic pressing of the charge under 56 MPa

The pressing predictor, which is a categorical variable, is varied from 0 to 1, where 0 means a low load of 0.7 MPa and 1 means a high load under 56 MPa.

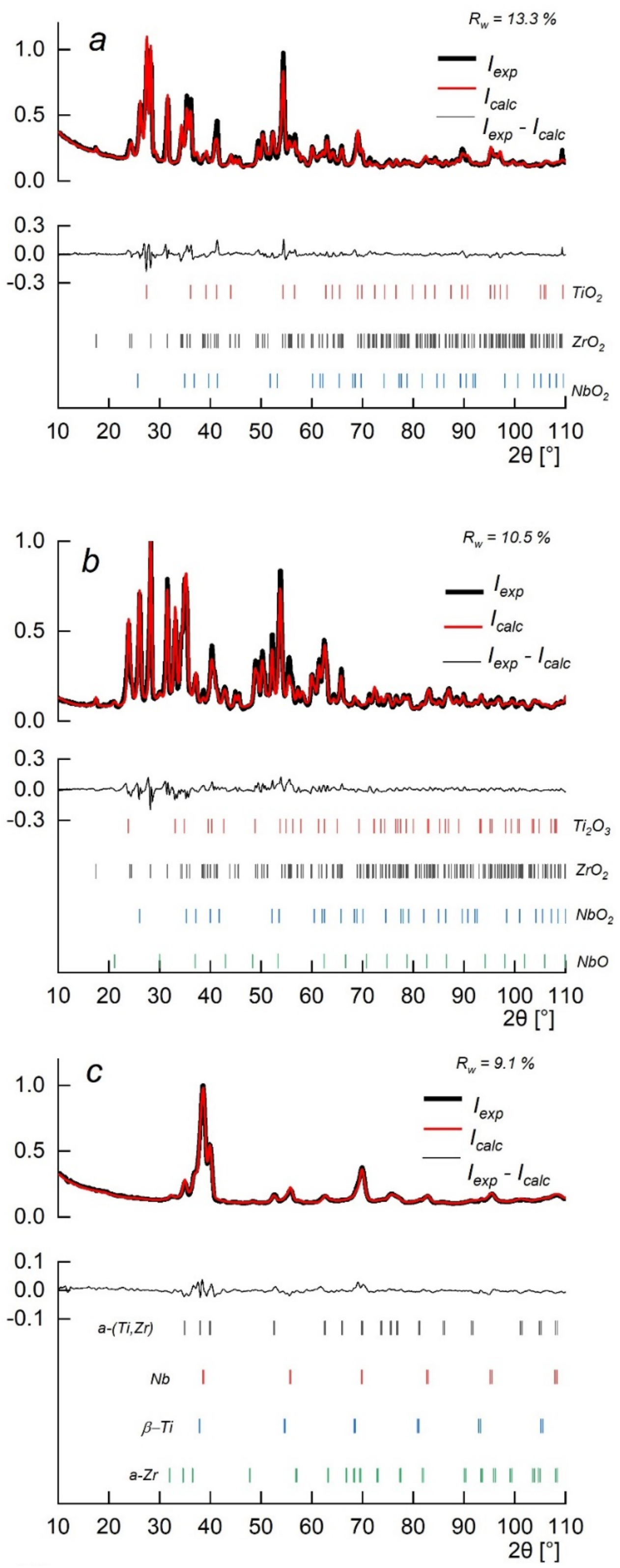

a
R_w = 13.3 %
I_{exp}
I_{calc}
I_{exp} - I_{calc}
TiO_2
ZrO_2
NbO_2
2θ [°]
b
R_w = 10.5 %
I_{exp}
I_{calc}
I_{exp} - I_{calc}
Ti_2O_3
ZrO_2
NbO_2
NbO
2θ [°]
c
R_w = 9.1 %
I_{exp}
I_{calc}
I_{exp} - I_{calc}
a-(Ti,Zr)
Nb
β–Ti
a-Zr
2θ [°]

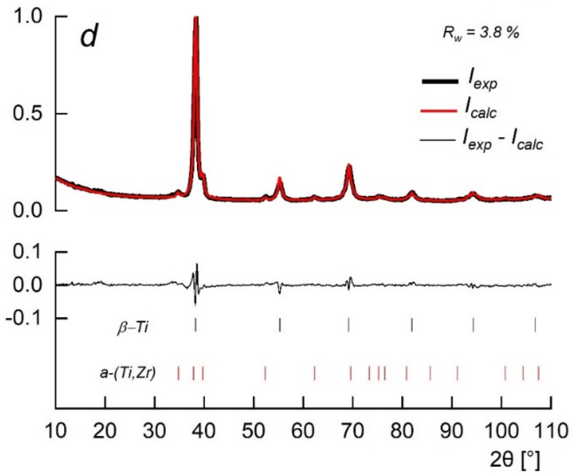

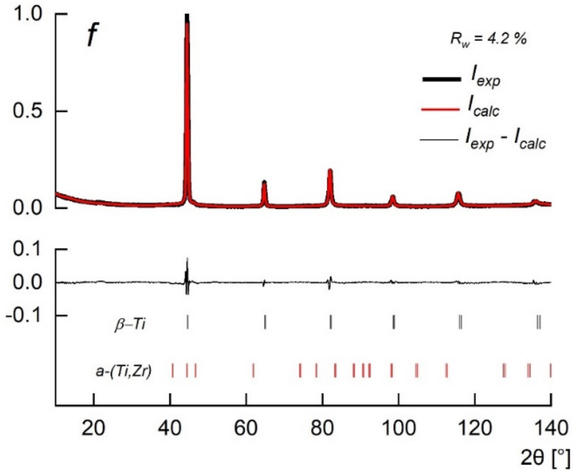

Figure S1. Experimental and calculated XRD patterns of calcium-hydride powders synthesized at various temperatures and exposure: *a* – 900 °C, 0 h; *b* – 1000 °C, 0 h; *c* – 1000 °C, 6h; *d* – 1200, 4 h; *f* – 1200, 12 h. *a, c, d, f* - (ρ_{charge} = 1.4 g/cm^3); *b* - (ρ_{charge} = 1.6 g/cm^3).
R_w – weighted average Bragg R-factor.

Table S2. Several structural parameters of the phases, which Bragg positions are shown in Figure S1.

Phase	Structure type (s.g.)	Lattice parameters	Phase content	
			Weight fraction, %	Volume fraction, %
Graph a)				
TiO_2	P 42/m n m (136)	a= 4.597<br>c= 2.961	65.7	72.2
ZrO_2	P 1 21/c 1 (14)	a= 5.138<br>b= 5.200<br>c= 5.309<br>β=99.226	25.4	20.7
NbO_2	P 42/m n m (136)	a= 4.838<br>c= 2.990	8.9	7.1
Graph b)				
ZrO_2	P 1 21/c 1 (14)	a= 5.140<br>b= 5.199<br>c= 5.310<br>β =99.196	31.5	27.8
Ti_2O_3	R -3 c (167)	a= 5.145<br>c= 13.655	53.3	59.2
NbO_2	P 42/m n m (136)	a= 4.838<br>c= 2.990	13.2	11.5
NbO	P m -3 m (221)	a= 4.207	2.1	1.5
Graph c)				
β-*Ti*	I m -3 m (229)	a= 3.345	11.3	10.3
Nb	I m -3 m (229)	a= 3.296	24.4	17.9
α-Zr	P 63/m m c (194)	a= 3.201<br>c= 5.133	7.8	7.6
α-(Ti,Zr)	P 63/m m c (194)	a= 2.966<br>c= 4.742	56.5	64.2
Graph d)				
β-*Ti*	I m -3 m (229)	a= 3.325	80.6	75.7
α-(Ti,Zr)	P 63/m m c (194)	a= 2.981<br>c= 4.754	19.4	24.6
Graph f)				
β-*Ti*	I m -3 m (229)	a= 3.343	94.9	93.2
α-(Ti,Zr)	P 63/m m c (194)	a= 2.978<br>c= 4.837	5.1	5.4

* The solid solution of Zr and Nb in Ti lattice is referred as β-*Ti* in the table and in the corresponding Figure S2; *α-(Ti,Zr*) indicates that there is the difference between a calculated lattice parameter and the parameter of pure α-Ti.

The calcium-hydride powders of Ti, Zr, and Nb possess a spongy-type morphology regardless of the morphology of initial oxides (TiO_2, ZrO_2, and Nb_2O_5) (Figure S2).

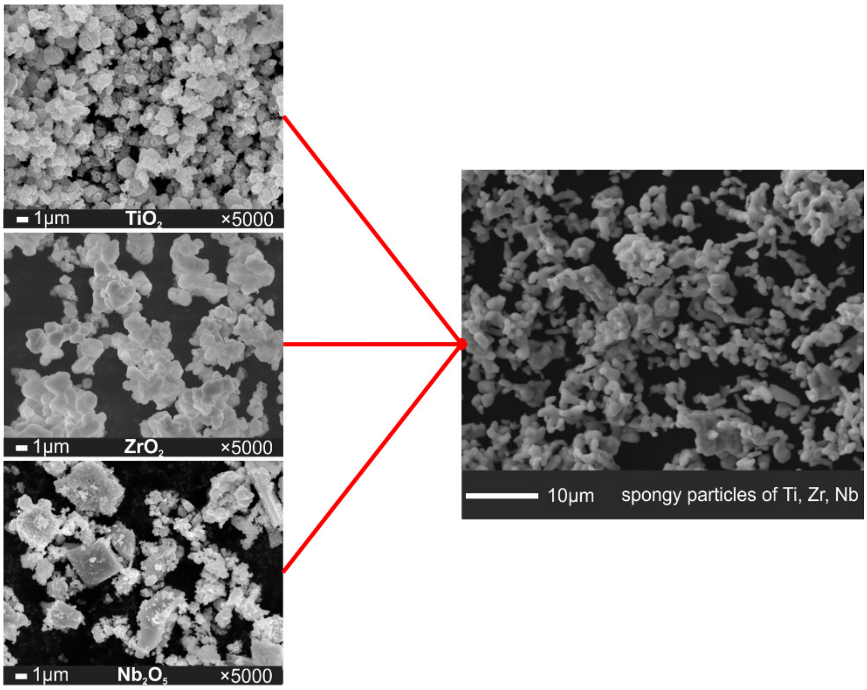

Figure S2. Typical morphology of Ti, Zr, and Nb reduced by calcium hydride.

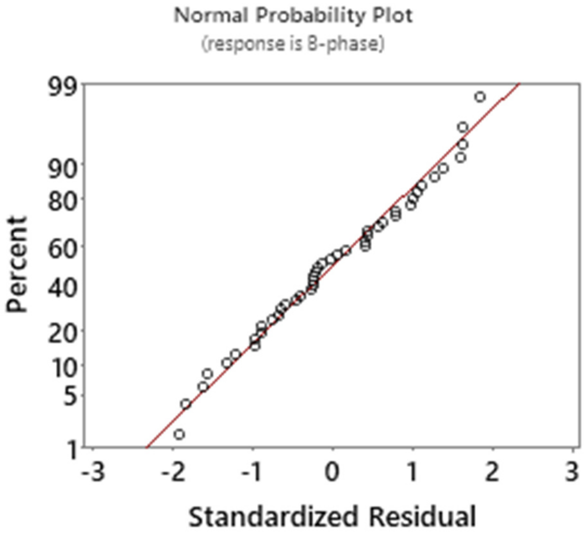

Figure S3. Normal probability plot of standardized residuals for the elaborated model.
